# Neutrophil-lymphocyte ratio predicts recurrence in patients with resected stage 1 non-small cell lung cancer

**DOI:** 10.1186/s13019-018-0763-0

**Published:** 2018-06-27

**Authors:** Shinjiro Mizuguchi, Nobuhiro Izumi, Takuma Tsukioka, Hiroaki Komatsu, Noritoshi Nishiyama

**Affiliations:** Department of Thoracic Surgery, Osaka City University Hospital, 1-4-3 Asahimachi, Abeno-ku, Osaka, 545-8585 Japan

**Keywords:** Non-small cell lung cancer, Prognosis, Recurrence-free survival, Neutrophil-lymphocyte ratio, Surgery

## Abstract

**Background:**

The aim was to determine the prognostic value of the neutrophil-lymphocyte ratio (NLR) in patients with completely resected stage 1 non-small cell lung cancer (NSCLC).

**Methods:**

The study enrolled 382 NSCLC patients, and an optimal NLR cutoff value was determined by ROC analysis. Patients were divided by preoperative NLR into low (< 1.5, *n* = 99), intermediate (1.5 ≤ NLR < 3.5, *n* = 245), and high (NLR ≥ 3.5, *n* = 38) value groups. Serum diacron-reactive oxygen metabolites (d-ROMs) were assayed in 33 consecutive patients and used as an indicator of oxidative stress.

**Results:**

The mean NLR in patients with high d-ROMs (> 300 U.CARR, *n* = 16) was 1.72 ± 0.67, which was significantly higher than that in patients with low d-ROMs (1.41 ± 0.39, *n* = 17; *P* = 0.018). The 3-, 5- and 10-year survival rates in the three NLR groups were 92, 77, and 59% (low); 82, 70, and 50% (intermediate); and 76, 58, and 32% (high) (*P* = 0.034). The 1-, 3- and 5-year recurrence-free survival rates in the three groups were 98, 90, and 86% (low), 91, 77, and 74% (intermediate); and 92, 77, and 68% (high) (*P* = 0.033). Multivariate analysis found that although NLR was not predictive of overall survival, high NLR was an independent risk factor of recurrence (hazard ratio: 2.03, 95% confidence interval: 1.17–3.79, *P* = 0.011) as were as age, pathological stage, tumor differentiation, and lymph-vascular invasion.

**Conclusions:**

A low preoperative NLR predicted good prognosis, and was associated with low systemic inflammation status in patients with stage 1 NSCLC. It may be helpful when considering intervals of routine follow-up or choice of adjuvant therapy.

## Background

Interest in links between systemic inflammation and the management of cancer is increasing. Many cancers develop at sites of infection, chronic irritation, and inflammation, and regardless of the location, inflammatory cells in the tumor microenvironment are indispensable participants in the neoplastic process, promoting cell proliferation, survival, angiogenesis, and migration [1]. Loss of tissue integrity caused by reduction of cellular adhesion is an early step in metastasis, allowing the spread of tumor cells from the primary tumor [2]. In mammary epithelial cells, malignant transformation and metastasis are stimulated by generation of endogenous reactive oxygen species (ROS) [3]. ROS contribute to carcinogenesis and the aberrant growth, metastasis, and angiogenesis that are characteristic of malignant tumors [4, 5] and associated with oxidative stress.

The evidence for neutrophil-lymphocyte ratio (NLR) as a novel marker of systemic inflammation, immunological response, and prognosis has been recently reviewed. A systematic review found that the NLR had independent prognostic value in unselected cohorts of more than 12,000 routinely treated patients, operative disease (20 studies), patients receiving neoadjuvant treatment and resection (5 studies), patients receiving chemo/radiotherapy (12 studies), and patients with inoperable disease (6 studies) [6, 7]. In advanced lung cancer patients, both the European Lung Cancer Working Group [8] and the Japan Multinational Trial Organization [9] reported that an elevated neutrophil count was an independent prognostic factor in patients with advanced non-small cell lung cancer (NSCLC). Several studies have evaluated the prognostic significance of the NLR in patients with completely resected NSCLC [10–17], but, the prognostic value of preoperative NLRs in early stage, completely resected NSCLC is not well known. The study objective was to determine the significance of increased NLR and its relationship to serum ROS generation, survival, and recurrence in patients with stage 1 NSCLC.

## Methods

### Patients

The medical records of 587 consecutive patients at Osaka City University Hospital, Osaka, Japan, with pulmonary resection for stage I NSCLC between January 1998 and December 2012 were analyzed retrospectively. Patients with partial wedge resection or segmentectomy, those without radical mediastinal lymph node dissection (R0), and those given neoadjuvant therapy were excluded from the study. Patients with hematologic cancers, autoimmune disorders, with recent steroid or immunosuppressive therapy, or preoperative infection were excluded from the NLR analysis. The records of the 382 remaining patients with pathological stage I NSCLC with lobectomy or bilobectomy and R0 were evaluated. Tumor histology was classified following World Health Organization criteria, and postoperative staging was based on the international TNM classification for lung cancer (7th) [18]. Patients were followed at 1–6-month intervals postoperatively. Follow-up evaluation included physical examination, chest X-ray, and blood examination, including for tumor markers. Chest, brain and abdominal computed tomography were performed at 6–12 month intervals. Bone scanning was not routinely performed in asymptomatic patients. Whenever any symptoms or signs of recurrence were detected, magnetic resonance imaging of the brain and bone scintigraphy was performed. Of the 382 patients, 264 had adenocarcinoma, 92 had squamous cell carcinoma, 12 had adenosquamous carcinoma, 14 had large-cell neuroendocrine carcinoma; 212 were pathological stage IA and 170 were stage IB. Fifty-one of 140 patients (36%) with stage 1A (T1b) and 1B adenocarcinoma received oral fluoropyrimidine for 2 years. Dosage escalation or schedule modification was at the discretion of the clinician. Patients underwent chemotherapy, radiotherapy, or the best available supportive care when recurrence was detected. This study was conducted following Helsinki Declaration guidelines and was approved by the institutional review board of Osaka City University (reference number 3361).

### NLR

Preoperative NLRs were calculated from routine blood counts performed on admission. The optimal NLR cutoff value for predicting recurrence within 3 years, as identified by receiver operating characteristic (ROC) curves, was 1.5 (Youden index = 0.154), the sensitivity was 30.6%, specificity was 84.8%, and area under the curve (AUC) = 0.572; 95% CI: 0.503–0.638). The patients were stratified by their preoperative NLR to three groups: low (< 1.5, *n* = 99), intermediate (1.5 ≤ NLR < 3.5, *n* = 245), and high (≥3.5, *n* = 38). The clinicopathological features, clinical course, and postsurgical survival of the groups were compared.

### Assay of reactive oxygen metabolites (ROM) in serum

Serum diacron (d)-ROM levels of 33 consecutive patients were measured as an indicator of oxidative using a spectrophotometric method using a commercial free radical analysis system (FRAS; Diacron, Grossto, Italy) as previously described [19]. As hydroperoxides are an intermediate oxidation product of lipids, peptides, and amino acids, overall oxidative stress can be spectrophotometrically estimated by measuring total hyperperoxide level [20]. Serum samples were collected just before surgery and stored at − 80 °C until they were assayed. Briefly, 10 μL of serum were added to 1 mL of assay mixture, gently agitated for 1 min at 37 °C, and the optical density (OD) was measured at 505 nm using a spectrophotometer. The results were expressed in Carratelli (CARR) units, where 1 U. CARR corresponds to 0.08 mg H_2_O_2_/100 mL serum [20].

### Statistical analysis

Values of continuous and dichotomous variables were compared using Kruskal−Wallis one-way analysis, the Mann−Whitney U test, the χ^2^ test, or Fisher’s exact test. The Kaplan−Meier method and log-rank test were used to analyze survival. To determine the independent prognostic factors, multivariate analysis was conducted using the Cox proportional hazard model. *P*-values < 0.05 were considered statistically significant. Statistical analysis was performed using JMP 10 software (SAS Institute, Cary, NC, USA).

## Results

### Relation between serum d-ROM and NLR

**Fig. 1:**
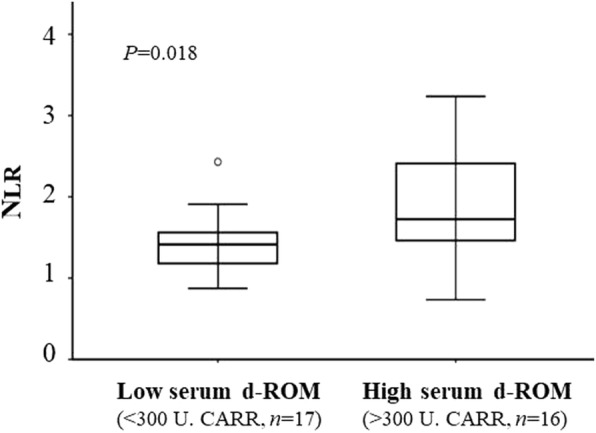
Distribution of NLR in individual patients with low and high serum d-ROM. Data are presented as upper and lower quartile range (box), median value (horizontal line), and middle 90% distribution (whisker line)

The mean d-ROM value of the 33 patients tested was 297 U.CARR (range, 215–434). As shown in Fig. 1, the mean NLR in patients with high (> 300 U.CARR, *n* = 16) d-ROM levels was 1.72 ± 0.67, which was significantly higher than that in patients with low (< 300 U.CARR, *n* = 17) d-ROM levels (1.41 ± 0.39, *P* = 0.018).

### Clinicopathological characteristics and NLR

Of the 382 patients, there were 264 with adenocarcinoma, 92 with squamous cell carcinoma, 12 with adenosquamous carcinoma, and 14 with large cell neuroendocrine carcinoma. Twelve patients were pathological stage IA, and 170 were stage IB. Table 1 shows the characteristics of patients stratified by NLR into low-, intermediate- and high-value groups). There were no significant differences in age, sex, smoking history, Eastern Cooperative Oncology Group performance status (PS), postoperative predicted pulmonary function tests (PFTs), differentiation of the resected tumor, pathological stage, Child-Pugh score, estimated glomerular filtration rate (eGFR), or serum albumen concentration among the groups. Patients with high NLRs had a significantly lower body mass index (BMI) and higher concentration of serum C-reactive protein (CRP) than those in the other two groups (*P* = 0.049 and < 0.001, respectively).

**Table 1:** Clinicopathological characteristics according to preoperative NLRs

Group		Patients	low NLRs	intermediate NLRs	High NLRs	*P*-value
			(< 1.5: *n* = 99)	(1.5–3.5: *n* = 245)	(≥3.5: *n* = 38)	
Age (years)	< 75	289	78	182	29	0.675
	≥75	93	21	63	9	
Sex	Male	232	57	154	21	0.509
	Female	150	42	91	17	
BMI (Kg/m^2^)			22.3 ± 3.0	22.3 ± 3.2	21.0 ± 3.0	0.049
Smoking History	Yes	240	61	156	23	0.895
	No	142	38	89	15	
PS	0–1	355	94	224	37	0.273
	2	27	5	21	1	
Predicted post PFTs^a^	≥40	355	95	227	33	0.184
	< 40	27	4	18	5	
Histology	Ad	264	79	166	19	0.003
	Others	118	20	79	19	
Differentiation	Well	125	37	78	10	0.412
	Mod/poor	257	62	167	28	
p-stage	IA	212	62	131	19	0.233
	IB	170	37	114	19	
Child-Pugh Score	5	342	90	220	32	0.539
	≥6	40	9	25	6	
eGFR (ml/min/1.73m^2^)	≥60	301	85	189	27	0.094
	< 60	81	14	56	11	
Albumin (g/dl)			4.1 ± 0.29	4.1 ± 0.35	4.1 ± 0.38	0.798
CRP (mg/dl)			0.14 ± 0.39	0.22 ± 0.43	0.87 ± 2.18	<0.001

^a^Predicted postoperative values of FEV_1.0_ or DL_CO_ less than 40% are defined as high-risk results of pulmonary function tests

*Ad* adenocarcinoma, *BMI* body mass index**,** *PFT* pulmonary function test, *PS* performance status, *p-stage* pathological stage

### Postoperative outcome

The overall mean duration of follow-up was 5.6 years (range 0.1–16.2 years), a total of 2146 patient-years. There were 63 deaths from lung cancer and 73 from other diseases, including 23 from other cancers; 246 patients were still alive, including 16 with a recurrence of lung cancer. Of the 99 patients with low NLRs, 10 (10%) died from lung cancer, 19 (19%) from other diseases, and 70 (71%) were still alive. Of the 245 patients with intermediate NLRs, 47 (19%) died from cancer, 43 (18%) from other diseases, and 155 (63%) were still alive. Of the 38 patients with high NLRs there were six deaths from cancer (16%), 11 from other diseases (29%) and 21 patients were still alive (55%). Patients with intermediate and high NLRs (i.e., ≥1.5) had a significantly greater risk of death not related to lung cancer than those with low NLRs (HR = 2.23, 95% CI; 1.18–4.66; *P* = 0.012).

There was no difference between the three groups in ratio of receiving chemotherapy or radiation therapy excluding palliative irradiation: Postoperative oral fluoropyrimidine was received in 26% (11/42) of low, 29% (26/89) of intermediate and 44% (4/9) of high NLRs (*P* = 0.570). After detection of lung cancer recurrence, systemic chemotherapy or radiation therapy were performed in 92% (11/12) of low, 77% (44/57) of intermediate and 56% (5/9) of high NLRs (*P* = 0.151).

**Fig. 2:**
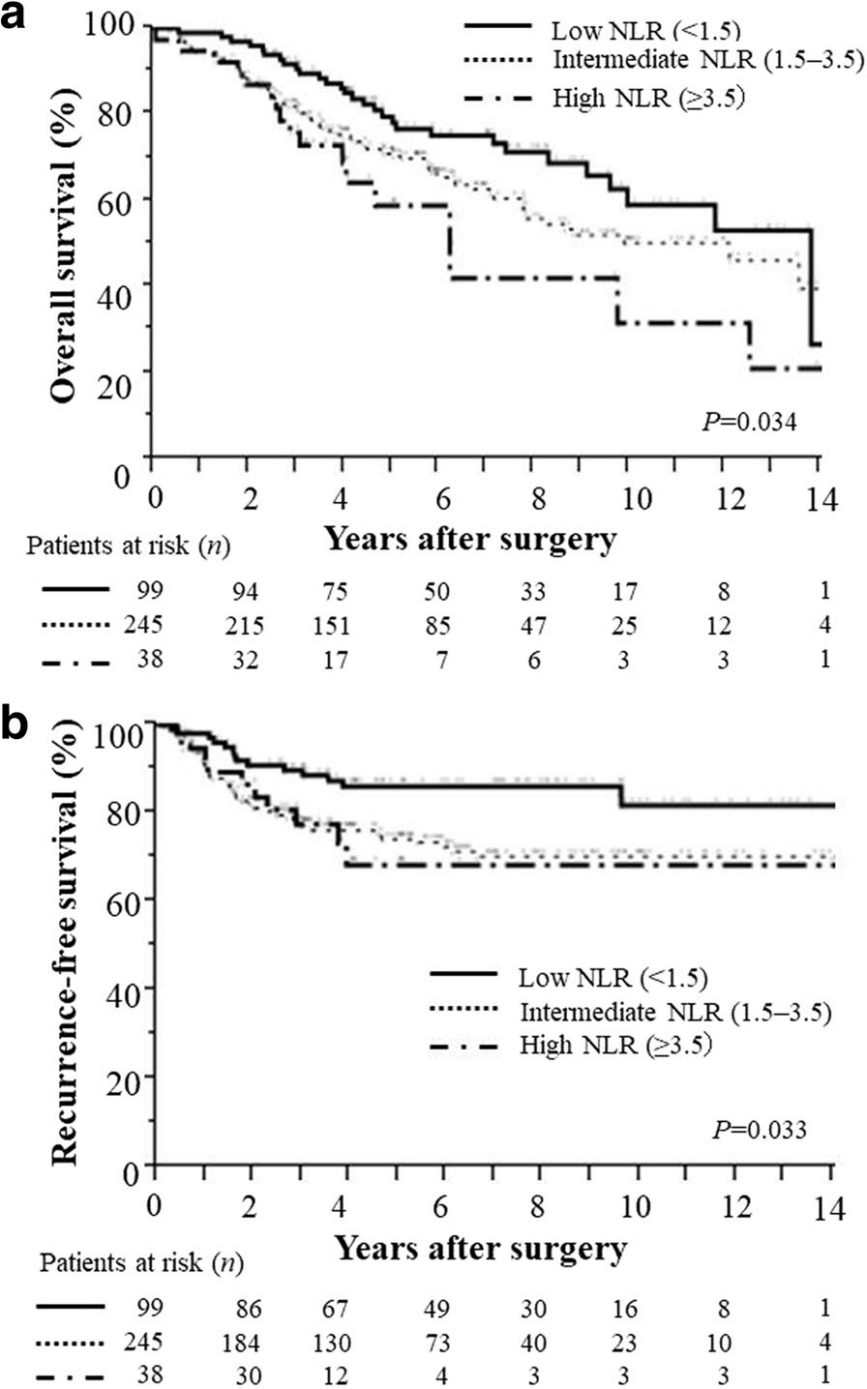
(**a**) Overall survival and (**b**) Recurrence-free survival of patients with resected stage 1 NSCLC according to the NLR. The overall survival rate and the recurrence-free survival rate of patients with low NLRs was significantly higher than that in patients with intermediate and high NLRs

As shown in Fig. 2a, the 3-, 5- and 10-year survival rates in patients with low, intermediate, and high NLRs were 92, 77, and 59%; 82, 70, and 50%; and 76, 58, and 32%, respectively (*P* = 0.034). The survival rate of patients with low NLRs was significantly higher than that of those with intermediate (HR =1.507, 95% CI: 1.004–2.331; *P* = 0.047) and high (HR = 2.133, 95% CI: 1.144–3.855, *P* = 0.018) NLRs. Regarding progression, recurrence-free survival is shown in Fig. 2b. The 1-, 3- and 5-year recurrence-free rates were 98, 90 and 86% in patients with low NLRs; 91, 77 and 74% in those with intermediate NLRs; and 92, 77, and 68% in those with high NLRs (*P* = 0.033).

### Multivariate analysis of NLR and clinicopathological variables

Univariate analysis and the log-rank test found that sex, age, PS, smoking history, NLR (> 1.5; intermediate, and high), postoperative PFTs, histology, degree of tumor differentiation, lymph-vascular invasion, pathological stage, postoperative complications, and some preoperative comorbidities (i.e., cardiac, cerebral, kidney and liver disease; and a prior history of tumors) were significantly associated with survival (Table 2). Multivariate analysis including the significant variables confirmed sex, age, PS, histology (non-adenocarcinoma), differentiation, lymph-vascular invasion, pathological stage, and a history of prior tumors as independent predictors of overall survival. NLR was not predictive of overall survival.

**Table 2:** Multivariate analysis of factors predicting overall survival

Factors	Univariate (*P*-value)	Multivariate (*P*-value)	Risk ratio	95% CI
Sex (male vs. female)	< 0.001	< 0.001	2.26	1.43–3.65
Age (≥70 years)	< 0.001	< 0.001	2.64	1.81–3.90
PS (2 vs 0–1)	< 0.001	0.026	2.04	1.10–3.56
Smoking	0.002	0.257	–	–
NLR (> 1.5)	0.028	0.316	–	–
Predicted post PFT^a^	0.025	0.058	–	–
Histology (other vs. adenocarcinoma)	0.034	0.021	1.60	1.07–2.41
Differentiation (m/p vs. well)	< 0.001	0.035	1.64	1.03–2.66
Lymph-vascular invasion	0.002	0.029	1.50	1.04–2.16
Pathological stage (IB vs. IA)	< 0.001	0.006	1.68	1.16–2.45
Postoperative complications	0.024	0.449	–	–
Preoperative comorbidities				
Hypertension	0.829	–	–	–
Diabetes mellitus	0.496	–	–	–
eGFR (< 70 mL/min/1.73 m^2^)	0.022	0.890	–	–
Child–Pugh classification (B or C)	0.028	0.880	–	–
Cardiac disease	0.011	0.301	–	–
Cerebral disease	0.014	0.876	–	–
Any prior tumors	< 0.001	< 0.001	3.37	2.27–4.96

^a^Predicted postoperative values of FEV_1.0_ or DLCO < 40% are defined as high-risk for PFTs

*PS* performance status, *NLR* neutrophil-lymphocyte ratio, *PFT* pulmonary function test, *eGFR* estimated glomerular filtration rate, *m/p* moderate or poor

The analysis of factors that increased risk of recurrence is shown in Table. 3. Univariate analysis and the log-rank test found that sex, age, smoking history, NLR > 1.5, tumor differentiation, lymph-vascular invasion, pathological stage, and preoperative cerebral comorbidity were significantly associated with recurrence. According to multivariate analysis, age, differentiation, lymph-vascular invasion, and pathological stage were independent predictors of overall survival. NLR (HR = 2.03, 95% CI: 1.17–3.79; *P* = 0.011) was a significant risk factor of recurrence as were age, pathological stage, differentiation of resected tumor, and lymph-vascular invasion.

**Table 3:** Multivariate analysis of factors predicting recurrence-free survival

Factors	Univariate (*P*-value)	Multivariate (*P*-value)	Risk ratio	95% CI
Sex (male vs. female)	0.001	0.118	–	–
Age (≥70 years)	< 0.001	< 0.001	2.20	1.38–3.53
PS (2 vs. 0–1)	0.254	–	–	–
Smoking	0.005	0.183	–	–
NLR(> 1.5)	0.009	0.011	2.03	1.17–3.79
Predicted post PFT^a^	0.211	–	–	–
Histology (other vs adenocarcinoma)	0.123	0.090	–	–
Differentiation (m/p vs. well)	0.004	0.038	1.77	1.03–3.18
Lymph-vascular invasion	< 0.001	< 0.001	2.31	1.47–3.66
Pathological stage (IB vs. IA)	< 0.001	0.003	2.09	1.27–3.48
Postoperative complication	0.368	–	–	–
Preoperative comorbidities				
Hypertension	0.846	–	–	–
Diabetes mellitus	0.797	–	–	–
eGFR (< 70 mL/min/1.73 m^2^)	0.107	0.647	–	–
Child–Pugh classification (B or C)	0.246	–	–	–
Cardiac disease	0.389	–	–	–
Cerebral disease	0.008	0.367	–	–
Any prior tumors	0.191	0.070	–	–

^a^Predicted postoperative values of FEV_1.0_ or DLCO < 40% is defined as high-risk for PFTs

*PS* performance status, *NLR* neutrophil-lymphocyte ratio, *PFT* pulmonary function test, *m/p* moderate or poor, *eGFR* estimated glomerular filtration rate

## Discussion

We found that an increase in the NLR was associated with systemic inflammation and predicted recurrence in patients with completely resected stage 1 NSCLC. We also found a positive relationship between serum ROS concentration and the NLR in those patients. Numerous physiological variables have been reported as markers of long-term survival following pulmonary resection for lung cancer. These include age, sex, PS, weight loss, sarcopenia, depressed mood, quality of life, smoking, arterial blood gases, Charlson Comorbidity Index score, forced expiratory volume in 1 s (FEV_1.0_), and diffusing capacity of the lungs for carbon monoxide (DLCO) [21–24]. The NLR is often used as an inflammation marker, and its prognostic value in lung cancer has been recently reported [7, 25–27]. The patients in this series with intermediate and high NLRs (i.e., ≥1.5) had a significantly greater risk of death not related to lung cancer than those with low NLRs.

Previous reports and meta-analyses [7, 25, 26] found that an elevated NLR was a marker of poor prognosis, and was associated with recurrence of lung cancer. In cancer patients, oxidative stress can be caused by various tumor progression mechanisms, such as malignant conversion; tumor cell survival, proliferation, chemo- and radio-resistance, invasion, angiogenesis, metastasis, and stem cell survival [4, 5] However, it is not possible to evaluate oxidative stress within the tumor microenvironment of living organs. Unlike previous studies that enrolled heterogeneous groups including patients with different NSCLC stages, we focused on patients with stage 1 disease. Tumor progression and/or tumor burden were thus limited, and patients with symptoms, treatments, or histories that could influence their inflammatory or nutrition status were excluded. The serum d-ROM results obtained in this study mainly reflected systemic inflammation, with a relatively small contribution by carcinoma-induced inflammation. In patients in good general condition, the level of systemic oxidative stress may correlate with oxidative stress associated with the tumor micro-environment, and vice versa. This oxidative stress-inflammation interaction may induce factors that promote recurrence and tumor progression.

Based on that hypothesis, we measured serum ROMs, an indicator of systemic inflammation, to reveal the relationship with NLRs. We have reported that preoperative serum ROM level was an independent predictive factor for nodal involvement in patients with clinical stage I lung adenocarcinoma [19]. The AUC was 0.763 (95% CI 0.625–0.902), and the ROC curve provided a prognostic cutoff value of approximately 300 U.CARR [19]. In this study, the mean NLR in patients with low ROMs (< 300 U.CARR) was 1.4, a significantly lower value than that in patients with high ROMs. In patients with NLRs less than 1.5, a relatively small proportion of lymphocytes would result in decreased inflammatory stress and less promotion of cancer progression. Important to note, most of patients of Low (< 1.5) NLR (92%) were received systemic chemotherapy after recurrence of cancer in this study, suggesting their good general condition. Overall, NLRs might have both physiological and oncological prognostic value.

An optimal NLR cutoff value of 5 has been used to define high preoperative inflammatory status [12, 26, 27]. However, only four patients in this study had an NLR greater than 5. Therefore we stratified the patients into three groups by the NLRs determined by ROC analysis and then assessed survival in each group. In particular, we focused on low NLRs in patients with completely resected stage 1 NSCLC. The significance of the NLR in early stage NSCLC has recently been reported in stage 1 patients with complete tumor resection [14] or treated with stereotactic radiation therapy [28]. As in our patient population, their NLR cutoff values (2.5 and 2.98) were lower than those reported in previous studies that enrolled stage I–III patients with surgical resection [25, 26].

The main cause of recurrence after potentially curative surgery might be the growth of micro-metastases which had been established prior to resection. In this study, an NLR > 1.5, reflecting a low peripheral lymphocyte count, predicted recurrence within 3 years. Although there was no significant relation between NLR and initial recurrence site (i.e., local or distant metastasis) in this study (data not shown), Takahashi et al. reported that the proportion of distant metastasis was higher in patients with high NLRs than in those with low NLRs [14]. Peripheral lymphocyte count has also been considered as an important marker of cancer progression and recurrence and as an independent prognostic factor in node-negative NSCLC associated with vascular invasion [15]. Moreover, low lymphocyte counts that accompany chemotherapy in patients with advanced tumors may indicate low treatment effectiveness, and low NLRs may indicate a good response to chemotherapy after detection of recurrence [29]. Furthermore, high NLRs have also been associated with infiltration of tumor by lymphocytes with low CD3+ and high CD5+ expression [30]. The presence and type of lymphocytes in the tumor microenvironment might be useful as a marker of improved therapeutic response to immunotherapy.

## Conclusion

In patients with completely resected stage 1 NSCLC, NLR was associated with systemic inflammation and predicted recurrence. Routine monitoring of the NLR may be useful when planning follow-up intervals and considering adjuvant therapy. Further investigation is needed to reveal the significance of relationships between perioperative NLRs in early lung cancer and the tumor microenvironment, inflammation, and host immunity.

## Abbreviations

AUC: area under the curve
BMI: body mass index
CRP: C-reactive protein
DLCO: diffusing capacity of the lungs for carbon monoxide
eGFR: estimated glomerular filtration rate
FEV_1.0_: forced expiratory volume in 1 s
NLR: neutrophil-lymphocyte ratio
NSCLC: non-small cell lung cancer
PFTs: pulmonary function tests
PS: performance status
ROMs: reactive oxygen metabolites
